# Safety Parameters of Quantum Molecular Resonance Devices During Thyroid Surgery: Porcine Model Using Continuous Neuromonitoring

**DOI:** 10.3389/fendo.2022.924731

**Published:** 2022-06-22

**Authors:** Hsin-Yi Tseng, Tzu-Yen Huang, Yi-Chu Lin, Jia Joanna Wang, How-Yun Ko, Cheng-Hsun Chuang, I-Cheng Lu, Pi-Ying Chang, Gregory W. Randolph, Gianlorenzo Dionigi, Ning-Chia Chang, Che-Wei Wu

**Affiliations:** ^1^ Department of Otorhinolaryngology-Head and Neck Surgery, International Thyroid Surgery Center, Kaohsiung Medical University Hospital, Kaohsiung Medical University, Kaohsiung, Taiwan; ^2^ Department of Otorhinolaryngology-Head and Neck Surgery, Kaohsiung Municipal Siaogang Hospital, Faculty of Medicine, College of Medicine, Kaohsiung Medical University, Kaohsiung, Taiwan; ^3^ Department of Anesthesiology, Kaohsiung Municipal Siaogang Hospital, Kaohsiung Medical University Hospital, Faculty of Medicine, College of Medicine, Kaohsiung Medical University, Kaohsiung, Taiwan; ^4^ Department of Anesthesiology, Kaohsiung Municipal Tatung Hospital, Kaohsiung Medical University Hospital, Faculty of Medicine, College of Medicine, Kaohsiung Medical University, Kaohsiung, Taiwan; ^5^ Division of Thyroid and Parathyroid Endocrine Surgery, Department of Otolaryngology—Head and Neck Surgery, Massachusetts Eye and Ear Infirmary, Harvard Medical School, Boston, MA, United States; ^6^ Division of General Surgery, Endocrine Surgery Section, Istituto Auxologico Italiano (IRCCS), Milan, Italy; ^7^ Department of Pathophysiology and Transplantation, Faculty of Medicine and Surgery, University of Milan, Milan, Italy

**Keywords:** thyroid surgery, intraoperative neuromonitoring (IONM), recurrent laryngeal nerve (RLN), quantum molecular resonance (QMR) devices, porcine model safety parameters

## Abstract

**Objectives:**

Quantum molecular resonance (QMR) devices have been applied as energy-based devices in many head and neck surgeries; however, research on their use in thyroid surgery is lacking. This study aimed to investigate the safety parameters of QMR devices during thyroidectomy when dissection was adjacent to the recurrent laryngeal nerve (RLN).

**Methods:**

This study included eight piglets with 16 RLNs, and real-time electromyography (EMG) signals were obtained from continuous intraoperative neuromonitoring (C-IONM). QMR bipolar scissor (BS) and monopolar unit (MU) were tested for safety parameters. In the activation study, QMR devices were activated at varying distances from the RLN. In the cooling study, QMR devices were cooled for varying time intervals, with or without muscle touch maneuver (MTM) before contacting with the RLN.

**Results:**

In the activation study, no adverse EMG change occurred when QMR BS and MU were activated at distances of 2 mm or longer from the RLNs. In the cooling study, no adverse EMG change occurred when QMR BS and MU were cooled in 2-second intervals or immediately after MTM.

**Conclusion:**

QMR devices should be carefully used when performing RLN dissection during thyroid surgery. According to the activation and cooling safety parameters in this study, surgeons can avoid RLN injury by following standard procedures when using QMR devices.

## Introduction

Hemostasis of the blood-rich thyroid gland is an important issue during thyroid surgery, and postoperative hematoma is a life-threatening complication that should be avoided as much as possible (1). As an equipment to assist hemostasis, many energy-based devices (EBDs) have been developed in the past 30 years and are widely used in thyroid surgery (2). High temperatures are unavoidable when using EBDs, and direct or indirect thermal energy can transfer to the recurrent laryngeal nerve (RLN) and result in thermal injury (3–5). RLN thermal injury causes more irreversible impairment of vocal cord movement than RLN mechanical injury, resulting in more voice disturbance (6, 7).

The quantum molecular resonance (QMR) device is an innovative EBD that has been safely and effectively applied in otolaryngology-head and neck surgeries such as phonomicrosurgery (8), tonsillectomy (9–11), oral surgery (12), and adenoidectomy (13). QMR devices are powered using electron energy quanta, which is generated by alternate current and high-frequency electron waves, and it is characterized by a well-defined dominant wave at 4 MHz, followed by waves at 8, 12 and 16 MHz with reduced amplitude (13, 14). As the electron energy quanta is delivered, cell molecular bonds in human tissue resonate, followed by subsequent bond breakage and a minimal temperature increase (13–16). The hemostasis of the QMR device is completed by triggering the denaturation of the protein fibrinogen to coagulate after breaking cell molecular bindings. The process also activates the physiological coagulation cascade without the need for necrotic plugs, which is contrary to methods that use other cauterization methods (13, 14). Vesalius Quantum (Telea Engineering, Vicenza, Italy) is a popular QMR system and was adopted in the current study. Two surgical instruments were most commonly used in head and neck surgery, including the QMR bipolar scissor (BS) and the QMR monopolar unit (MU). Details about Vesalius Quantum can be found at https://teleamedical.com/en/vesalius-quantum).

QMR devices have been applied as energy-based devices in many head and neck surgeries; however, research on their use in thyroid surgery and dissection adjacent to the recurrent laryngeal nerve (RLN) is lacking. The purpose of this study was to investigate real-time electromyographic (EMG) data for the RLN to define safety parameters for using a QMR device in thyroidectomy. For this purpose, we recorded the dynamic laryngeal EMG signals under continuous intraoperative neuromonitoring (C-IONM), when the QMR device was activated at various distances from the RLN in the activation study, and when the QMR device was cooled after activation for various durations with or without a muscle touch maneuver (MTM) in the cooling study.

## Materials and Methods

### Animal Preparation and Anesthesia

This prospective porcine experimental study was performed by the IONM research team at Kaohsiung Medical University, Taiwan, which has a well-established C-IONM protocol for RLN research. The animal-use protocols comply with national/international regulations. Guidelines for animal experiments, including principles of replacement, reduction, and refinement, were strictly followed. This experimental study was also approved by the Institutional Animal Care and Use Committee of Kaohsiung Medical University, Taiwan (IACUC Approval No. 110129).

Anesthesia was initiated by intramuscular administration of 2 mg/kg tiletamine/zolazepam 30 minutes before the experiment. No muscle relaxants were used during anesthesia to avoid neuromuscular blockade, which could interfere with EMG signals during neuromonitoring. Endotracheal tube surface electrodes were used to record EMG signals. After the piglets were intubated, the tidal volume was set at 8–12 mL/kg, and the respiratory rate was set at 15 to 20 breaths per minute. General anesthesia was maintained with 1% to 2% sevoflurane.

### IONM Equipment Setting and Operation

**Figure 1:**
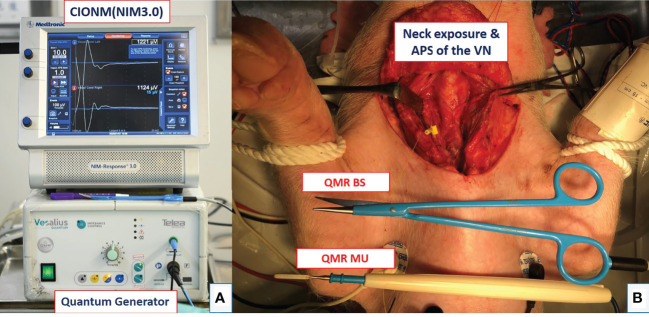
Equipment setting and animal setting. **(A)** Setup of the continuous intraoperative neuromonitoring (C-IONM) system with the Nerve Integrity Monitor (NIM 3.0) system and Quantum generator system (MX 90, Telea Engineering, Vicenza, Italy). **(B)** Neck wound exposure with automated periodic stimulation (APS) placed on the vagus nerve (VN). The quantum molecular resonance (QMR) bipolar scissor (BS) and monopolar unit (MU) are shown.

All piglets were placed in the supine position and intubated with a nerve integrity monitor size #6 EMG endotracheal tube (NIM Trivantage Tube, Medtronic, Jacksonville, Florida, USA). The EMG endotracheal tube as the recording electrode was applied to the piglets in the same conventional manner that it is applied to humans. The C-IONM monitoring system was performed by the Nerve Integrity Monitoring system (NIM 3.0, Medtronic, Jacksonville, Florida, USA) (**Figure 1A**). The automatic periodic stimulation (APS) as the stimulation electrode was placed on the vagus nerve (VN) (**Figure 1B**), and the nerve was stimulated continuously at 1 mA every second. The dynamic EMG signal changes during RLN injury were recorded efficiently and in real time.

The animal operation procedure began with a long transverse cervical incision, and the subcutaneous tissue and muscles were retracted away from the midline. The lateral border of the sternocleidomastoid (SCM) muscle was dissected and retracted medially. The thyroid gland, VNs, and RLNs were adequately exposed (**Figure 1B**). Next, RLNs were dissected and freed from fascia and kept dry for further experimental procedures. The APS was also set up at the fifth tracheal ring level with optimal stability. The amplitude and latency of the evoked response from VNs were calibrated to baseline values. Adverse EMG changes were defined as a 50% decrease in amplitude or a 10% increase in latency. Loss of signal (LOS) was defined as the amplitude of the RLN signal decreasing to less than 100 µV.

### Study Design

There are two studies in this experiment. The activation study determined the distance from the RLN at which the QMR devices could be safely activated. The cooling study determined the time intervals and procedures required to cool the QMR devices before further use for hemostasis and dissection near the RLN. **Figure 1A** shows the QMR Generator system (MX 90, Telea Engineering, Vicenza, Italy), and the power was set at 5 in this study. The QMR BS and MU instruments are shown in **Figure 1B**.

#### Activation Study

**Figure 2:**
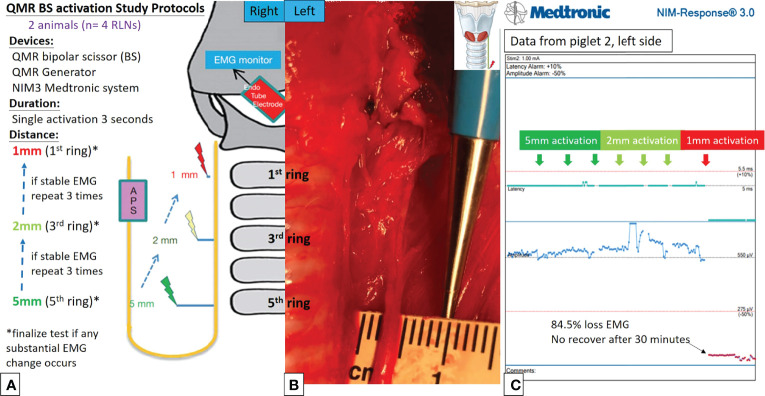
QMR BS activation study protocols. **(A)** Flowcharts of the QMR BS activation study. **(B)** QMR BS was tested at a distance of 5 mm from the left RLN. **(C)** EMG recording of the left side RLN in Piglet 2 under C-IONM. The QMR BS was activated at distances of 5, 2, and 1 mm. After activation at 1 mm, the EMG showed sudden 84.5% EMG signal loss without recovery after 30 minutes of observation.

**Figure 3:**
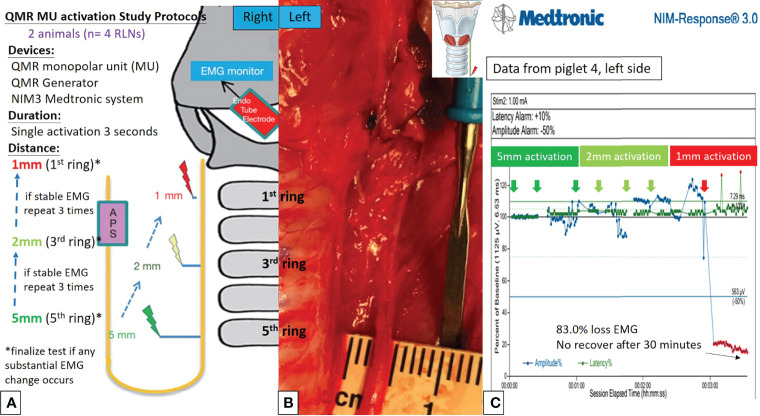
QMR MU activation study protocols. **(A)** Flowcharts of the QMR MU activation study. **(B)** QMR MU was tested at a distance of 5 mm from the left RLN. **(C)** EMG recording of the left side RLN in Piglet 4 under C-IONM. The QMR MU was activated at distances of 5, 2, and 1 mm. After activation at 1 mm, the EMG showed a sudden 83.0% EMG signal loss without recovery after 30 minutes of observation.

**Figures 2A, 2B**, **3A, 3B** describe the designs of the QMR BS and QMR MU activation studies, respectively. Both devices were applied to soft tissue in a single activation for 3 seconds. The QMR device activations began with a distance of 5 mm from the RLN. If there was no adverse EMG event (e.g., significant adverse decrease in amplitude or increase in latency) after 3 repetitions, the distance was progressively decreased to 2 mm (3 repetitions) and then to 1 mm (3 repetitions). Real-time EMG information under C-IONM was continuously recorded during each QMR device activation. If an adverse EMG event occurred, the RLN was interpreted as injured, and the procedure ended. Dynamic EMG changes were continuously recorded for at least 30 minutes to observe any recovery of the electrophysiological response.

#### Cooling Study

**Figure 4:**
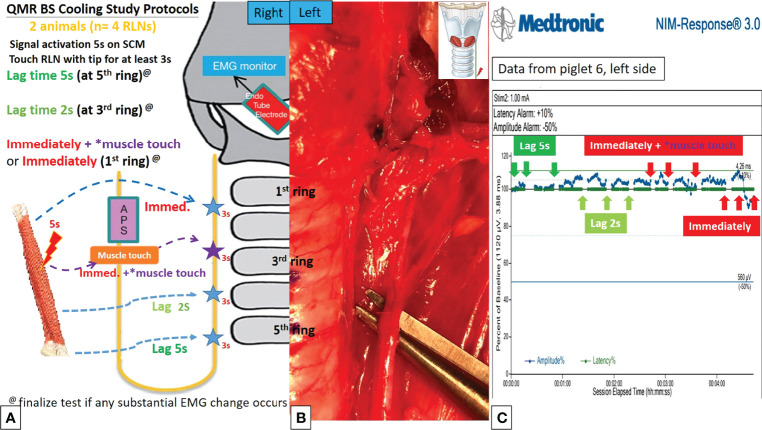
QMR BS cooling study protocols. **(A)** Flowchart of the QMR BS cooling study. **(B)** The open blade of QMR BS touched the left RLN. **(C)** EMG recording of the left side RLN in Piglet 6 under C-IONM. After a single activation on sternocleidomastoid (SCM) muscle, the cooling study was performed with 5 seconds, 2 seconds, immediately without muscle touch maneuver (MTM), and immediately with MTM. The EMG results showed no adverse effects. The EMG signal remained stable in repeated tests.

**Figure 5:**
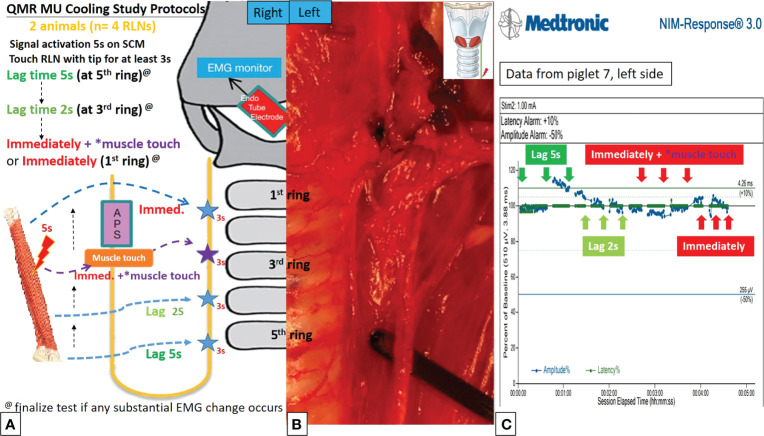
QMR MU cooling study protocols. **(A)** Flowchart of the QMR MU cooling study. **(B)** The blade of the QMR MU touched the left RLN. **(C)** EMG recording of the left side RLN in Piglet 7 under C-IONM. After a single activation of the SCM muscle, the cooling study was performed for 5 seconds, 2 seconds, immediately without MTM, and immediately with MTM. The EMG results showed no adverse effects. The EMG signal remained stable in repeated tests.

**Figures 4A, 4B**, **5A, 5B** describe the designs of the QMR BS and QMR MU cooling studies, respectively. The QMR device was applied to the SCM muscle in a single activation of 5 seconds followed by cooling for 5 seconds at room temperature. The blade of the QMR MU or the open blade of the QMR BS was then touched to the RLN for 3 seconds. If there was no adverse EMG event after 3 repetitions, the cooling time was decreased to 2 seconds, then immediately with “muscle touch maneuver (MTM)”, and then immediately without MTM. MTM means that after a single activation of the SCM muscle and before contact with the RLN, the operator quickly contacts the QMR device (blade of QMR MU and open blade of QMR BS) with the ipsilateral strap muscle to achieve a contact cooling effect. Real-time EMG information under C-IONM was continuously recorded during the cooling procedure of each QMR device. If an adverse EMG event occurred, the RLN was interpreted as injured, and the procedure ended. Dynamic EMG changes were continuously recorded for at least 30 minutes to observe any recovery of the electrophysiological response.

## Results

The animal anesthesia, surgical approach, and C-IONM of the RLNs were successfully performed in all studies.

### Activation Study

Activation study was performed in 8 RLNs of 4 piglets (animal No. 1-4). No adverse EMG events (amplitude decrease or latency increase) were observed when the QMR BS and QMR MU were activated at distances of 5 and 2 mm. With the QMR BS activated at a distance of 1 mm, all RLNs showed a signal decrease (63.0%, 60.0%, 84.5% loss and LOS) after a 30-minute observation (**Table 1**). The left RLN in piglet 2 showed 84.5% loss without recovery (**Figure 2C**). With the QMR MU activated at a distance of 1 mm, 3 of 4 RLNs showed a signal decrease (83.0% loss and 2 LOS) after a 30-minute observation (**Table 1**). The left RLN in piglet 4 showed 83.0% loss without recovery (**Figure 3C**).

**Table 1:** Activation study: real-time EMG changes after QMR BS and QMR MU activation at varying distances to the RLN.

Animal No.	Side	5mm, amplitude [times]	2mm, amplitude [times]	1mm, amplitude [times]
**1 (QMR BS)** **2 (QMR BS)** **3 (QMR MU)** **4 (QMR MU)**	Left Right Left Right Left Right Left Right	Stable [3] Stable [3] Stable [3] Stable [3] Stable [3] Stable [3] Stable [3] Stable [3]	Stable [3] Stable [3] Stable [3] Stable [3] Stable [3] Stable [3] Stable [3] Stable [3]	63.0% loss [1] 60.0% loss [1] 84.5% loss [1] LOS [1] Stable [3] LOS [1] 83.0% loss [1] LOS [1]

EMG, electromyographic; QMR, Quantum molecular resonance; BS, bipolar scissor; MU, monopolar unit; RLN, recurrent laryngeal nerve; LOS, loss of signal.

### Cooling Study

Cooling study was performed in 8 RLNs of 4 piglets (animal No. 5-8). In the QMR BS cooling study, no adverse EMG events were noted at any cooling time (5 seconds, 2 seconds, immediately with MTM, and immediately without MTM) (**Table 2**). The left RLN in piglet 6 showed a stable EMG signal without amplitude decrease or latency increase (**Figure 4C**).

**Table 2:** Cooling study: real-time EMG changes after QMR BS and QMR MU activation and varying cooling time.

Animal No.	Side	5 seconds, amplitude [times]	2 seconds, amplitude [times]	Immediately with MTM, amplitude [times]	Immediately without MTM, amplitude [times]
**5 (QMR BS)** **6 (QMR BS)** **7 (QMR MU)** **8 (QMR MU)**	Left Right Left Right Left Right Left Right	Stable [3] Stable [3] Stable [3] Stable [3] Stable [3] Stable [3] Stable [3] Stable [3]	Stable [3] Stable [3] Stable [3] Stable [3] Stable [3] Stable [3] Stable [3] Stable [3]	Stable [3] Stable [3] Stable [3] Stable [3] Stable [3] Stable [3] Stable [3] Stable [3]	Stable [3] Stable [3] Stable [3] Stable [3] Stable [3] LOS [1] Stable [3] LOS [1]

EMG, electromyographic; QMR, Quantum molecular resonance; BS, bipolar scissor; MU, monopolar unit; LOS, loss of signal.

In the QMR MU cooling study, there were no adverse EMG events when the cooling time was more than 2 seconds or immediately with MTM. When the cooling time was immediately without MTM, 2 of 4 RLNs with LOS after 30 minutes of observation (**Table 2**). The left RLN in piglet 7 showed a stable EMG signal (**Figure 5C**).

## Discussion

The current study is the first to provide RLN safety parameters when using QMR devices in thyroid surgery. In the activation study, activation of the QMR BS and QMR MU at a distance longer than 2 mm from the RLN showed no adverse EMG events. In the cooling study, cooling of the QMR BS and QMR MU for 2 seconds or with MTM showed no adverse EMG events.

The application of EBDs during thyroid surgery has increased worldwide due to significantly reduced intraoperative blood loss, operating time, and postoperative hematoma rates (2, 17). In addition, the risk of thermal injury to nerves during thyroid surgery continues to increase (18). Sixty degree Celsius is a critical temperature, and exceeding this temperature will cause functional damage to the endoneurium (19, 20). Thermal injuries to nerves often occur suddenly and unexpectedly, and they are difficult to recognize visually and difficult to recover. Therefore, in addition to referring to the data provided by the manufacturer before using the new EBDs for thyroid surgery, a well-established C-IONM animal experimental model is helpful for safe application in clinical settings. Several animal studies have reported commonly used EBDs and their safety parameters during thyroidectomy. The safety parameters for devices such as the bipolar electrocautery, LigaSure, Harmonic scalpel, THUNDERBEAT, and FM devices were more than 2 mm to the nerve when activated, and a with 5 mm distance was best for monopolar electrocautery (21–27). All devices should be cooled by the MTM before contact with the RLN. The QMR MU and QMR BS are suggested to activate at 2 mm or longer from the RLN. Although the QMR BS showed a faster cooling time than QMR MU (less than the time the instrument required to move from the SCM muscle to the RLN), considering that the instruments are usually closed to the RLN during dissection, a MTM and an adequate cooling time (at least two seconds) are still essential cooling procedures that cannot be omitted.

The first experimental study of QMR energy involved performing a thoracotomy in rats and evaluating its effect histologically in 2007 (28). By resonating and breaking molecular boundaries, QMR devices are said to cut tissue while maintaining a low temperature around the tissue (29), and tissue dissection is not facilitated through thermal vaporization as it is with traditional electrocautery or lasers (13, 15). Some literature (13, 14) and the manufacturer’s website report that the temperature can be lower than 50 degrees Celsius with minimal thermal spread depth. D’Eredità et al. reported a mean 43 ± 9 µm depth of injury with QMR, while the depth of injury was 126 ± 11 µm with coblation in the histopathological evaluation of tonsillectomy specimens (11). For hemostasis, unlike electro and radiosurgery devices that produce a relatively large-area of burnt vessel collapse, QMR devices deploy a special waveform (with a slight loss of resonance) that elevates the temperature to 45-65°C (12, 28). This waveform triggers the denaturation process of fibrinogen, which turns into fibrin and clots the blood without causing thermal damage to the blood vessels. The above contents fully illustrate the potential benefits of QMR devices used in thyroid surgery, such as low temperature, effective hemostasis, and a small range of lateral thermal spread. However, in the dry environment, the QMR device reached a maximum temperature of 72°C after a single activation (3 seconds), and need approximately 2 seconds to decrease the temperature below 60°C in our preliminary thermographic study. It must be emphasized that, according to the results of this study, thyroid surgeons must still maintain adequate activation distance and cooling time to ensure the safety of their clinical application.

For the application of QMR devices in thyroid surgery, the interchangeability between QMR BS and QMR MU is an advantage worth mentioning. Thyroid surgeons can choose appropriate instruments according to the requirements of the thyroid dissection. In some relatively dense structures, such as Berry’s ligaments, the scissor structure of QMR BS can have advantages during dissection compared with the sealer structure of other EBDs. Hence, QMR instruments are more likely to be dissected when they are directly adjacent to the RLN, and knowledge of the neural safety parameters of QMR devices is even more necessary for thyroid surgeons.

Several limitations in the study need to be mentioned. First, this study only had a 30-minute observation period after an adverse EMG event, and nerves with incomplete signal loss may not predict postoperative neural function. However, because of the difficult-to-repair nature of thermal injury, the occurrence of adverse EMG events is a well-established way to assess the neural safety parameters of a new EBD (21–27). Second, some aspects in this prospective porcine model study may not be applicable to human surgery. Several factors can affect heat transfer from the QMR devices to the RLNs, including the difference in body temperature between human and experimental animals and the temperature of the surrounding environment. However, animal studies still provide useful and reliable data for human surgery. Finally, to have stable contact between the QMR devices and RLNs to avoid motion-affected C-IONM recordings, the part of the QMR devices that make direct contact with RLNs is closed but not the exact same as that in clinical surgery, especially the QMR BS cooling study. Therefore, although this study showed that QMR requires a very short cooling time, it is still recommended that the MTM or a 2-second cooling time should be performed before neural dissection.

## Conclusion

QMR devices should be carefully used when performing RLN dissection during thyroid surgery. Safety recommendations regarding the use of QMR MU and QMR BS are as follows: activation should be at least 2 mm from the RLN and cooling procedures should include the MTM or an adequate cooling time (at least two seconds). According to the activation and cooling safety parameters in this study, surgeons can avoid RLN injury by following standard procedures when using QMR devices.

## Data Availability Statement

The original contributions presented in the study are included in the article/supplementary material. Further inquiries can be directed to the corresponding author.

## Ethics Statement

The animal study was reviewed and approved by Institutional Animal Care and Use Committee of Kaohsiung Medical University, Taiwan (IACUC Approval No. 110129).

## Author Contributions

Supervision – GR, GD, N-CC, and C-WW; Materials – H-YT, T-YH, Y-CL, JW, H-YK, C-HC, and C-WW; Data Collection and Processing – H-YT, T-YH, N-CC, and C-WW; Analysis and Interpretation- H-YT, T-YH, I-CL, and P-YC; Literature Search - H-YT, T-YH, Y-CL, JW, H-YK, and C-HC; Writing Manuscript – All authors. All authors have read and agreed to the published version of the manuscript. All authors contributed to the article and approved the submitted version.

## Funding

This study was supported by grants from Kaohsiung Medical University Hospital, Kaohsiung Medical University (KMUH110-0R51), and Ministry of Science and Technology (MOST 110-2314-B-037-104-MY2, MOST 110-2314-B-037-120), Taiwan.

## Conflict of Interest

The authors declare that the research was conducted in the absence of any commercial or financial relationships that could be construed as a potential conflict of interest.

## Publisher’s Note

All claims expressed in this article are solely those of the authors and do not necessarily represent those of their affiliated organizations, or those of the publisher, the editors and the reviewers. Any product that may be evaluated in this article, or claim that may be made by its manufacturer, is not guaranteed or endorsed by the publisher.
